# Microvascular maturation of the septal capillary layers takes place in parallel to alveolarization in human lungs

**DOI:** 10.1152/ajplung.00425.2022

**Published:** 2023-08-22

**Authors:** Lukas Schmid, Dallas M. Hyde, Johannes C. Schittny

**Affiliations:** ^1^Institute of Anatomy, University of Bern, Bern, Switzerland; ^2^California National Primate Research Center, University of California, Davis, California, United States; ^3^Department of Anatomy, Physiology, and Cell Biology, School of Veterinary Medicine, University of California, Davis, California, United States; ^4^Center for Health and the Environment, University of California, Davis, California, United States

**Keywords:** human, lung maturation, microvascular maturation, postnatal lung development, stereology

## Abstract

Primary and secondary septa formed during lung development contain a double-layered capillary network. To improve gas exchange, the capillary network is remodeled into a single-layered one, a process that is called microvascular maturation (MVM). It takes place during classical and continued alveolarization. Classical alveolarization is defined as a formation of new septa from immature septa and continued alveolarization as a formation from mature septa. Until now, MVM was never quantitatively evaluated in human lungs. To correlate alveolarization and MVM, and to determine the transition point from classical to continued alveolarization, the degree of MVM was stereologically estimated. In 12 human lungs (0.1–15 yr), the alveolar surface area of immature and mature septa was estimated stereologically by intersection counting. An MVM-quotient (R_MVM_) was defined as the mature alveolar surface area over total alveolar surface area. The MVM-quotient increased logarithmically over age and showed a biphasic increase similar to alveolarization. It did not reach 100% maturity in these samples. A linear correlation between the MVM-quotient and the logarithm of the number of alveoli was observed. We conclude that MVM increased logarithmically and biphasically in parallel to alveolarization until alveolarization ceased. However, at 2–3 yr of age three-quarters of the alveolar microvasculature are mature. This result may explain a previous postulate that MVM is finished at this age. We hypothesize that as long as alveolarization takes place, MVM will take place in parallel. We propose that the transition from classical to continued alveolarization takes place between the ages of 1–3 yr in humans.

**NEW & NOTEWORTHY** Newly formed alveolar septa contain a double-layered capillary network. To optimize gas exchange, the two layers fuse to a single-layered capillary network during microvascular maturation. Because its timing is unknow in humans, microvascular maturation was stereologically estimated throughout postnatal human lung development. It is shown that maturation of the microvascular and alveolar septa takes place in parallel to alveolarization. At an age of 2–3 yr three-quarters of the septa are mature.

## Graphical abstract

**Figure:**
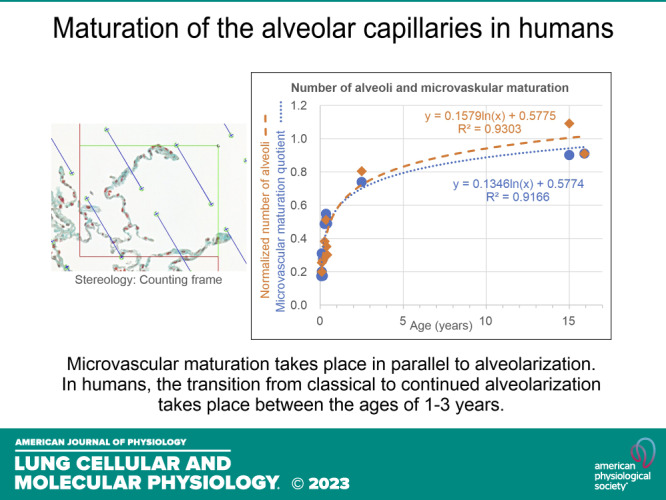

## INTRODUCTION

During the past 15 years, the development of new approaches like counting the alveoli (1), estimating the length of the free septal edge (2, 3), and high-resolution imaging (2, 4–6) has led to a better structural understanding of lung development. This was especially true for the phase of alveolarization. In 2014, Herring et al. (7) estimated the number of alveoli during human lung development. They showed an exponential increase until the age of 2 yr and a significantly slower one afterward. This result confirmed that earlier findings in rhesus monkeys (8), rats (2, 9), and mice (3) are also applicable to humans. Using hyperpolarized helium-3 (^3^He) magnetic resonance imaging to assess the alveolar size, Narayanan et al. (10) presented the first evidence that in humans, alveolarization continues during childhood and adolescence.

Starting shortly before birth in humans [36 wk after conception, 38 wk of pregnancy (11)], the process of alveolarization, meaning the building of new alveoli, represents the last phase of lung development. Alveolarization may be subdivided into two phases, classical and continued alveolarization. In classical alveolarization, new alveolar septa are built from preexisting, primary, or immature alveolar septa. The new septa subdivide the existing airspaces and increase the total surface area used for gas exchange. The primary or newly formed, still immature alveolar septa contain a double-layered capillary network. During microvascular maturation, the immature, double-layered capillary network of the alveolar septa fuses into a mature, single-layered capillary network, improving gas exchange and efficiency of the lung parenchyma (12). In continued alveolarization, new alveolar septa lift off from already mature alveolar septa with a single-layered capillary network, but a second capillary layer is built by angiogenesis instantly. Therefore, the new alveolar septa, built during continued alveolarization, possesses also a double-layered capillary network (2, 13). Originally, microvascular maturation was described as a process, following alveolarization and ending at the age between 2 and 3 yr in humans (12, 14). As it was shown in rats and mice that alveolarization and microvascular maturation of the alveolar septa takes place in parallel (15, 16), the same was proposed for humans (13). To evaluate this hypothesis and to identify the point of transition from classical to continued alveolarization, we estimated the maturation of the alveolar microvasculature in human lungs.

## MATERIALS AND METHODS

### Tissues

The human lung tissue was donated by D. M. Hyde (Department of Anatomy, Physiology, and Cell Biology, School of Veterinary Medicine, University of California, Davis). All specimens were coded, and the identifiers are not available anymore to the authors. Only the pertinent data published in Herring et al. (7) were available to the research personnel, the identifiers were not available to the measurers. The group of Dallas Hyde obtained the lung tissue from autopsies performed at the Sacramento County Coroner’s Office. The Research Review Committee of the Sacramento County Coroner’s Office approved the study. The study was determined to be exempted by the University of California, Davis, Committee on Human Research.

Herring et al. (7) weighed the removed lungs at the time of postmortem examination and a cannula was inserted in each mainstem bronchus and secured in position. With a fluid pressure of 25 to 30 cm, the lungs were inflated to full size with 10% neutral-buffered formalin for 15—30 min. After inflation, the lungs were suspended in a container containing identical fixative. Later, the lungs were removed from the fixative and lung volume was measured by water displacement. After volume measurement, the lung tissue was cut into sagittal slices of 10-mm thickness. Forensic pathologists examined all the slices to identify lesions in the lungs. Only lungs that did not show gross lesions were included in the study. Of the initially sagittal slices, strips of 10-mm width were cut and lined up according to tissue surface size, smallest to largest. Performing the smooth fractionator method (17) every other bar was selected and arranged in a horseshoe-shaped cycle. Starting on the end of the smallest and working to the other end, the first fraction was selected. The first fraction comprised ∼10 strips. The whole number of strips was divided by the desired number of strips to be sampled, giving the sampling interval. Starting on a random number between one and the total number of strips and counting every fraction thereof, the first strips were chosen. To gather the second fraction, the obtained strips were cut into smaller bricks of 1.5 cm and arranged in the same manner as the tissue strips above in a horseshoe-shaped ring, resulting again in a smooth fractionator. The desired number of bricks was defined as eight, giving the sampling interval of the whole number of bricks divided by eight. Again, starting on a random starting brick and advancing with the selecting interval, the final tissue blocks were selected. The tissue not selected by Herring et al. (7) during the application of the second smooth fractionator was sent to the authors’ laboratory preserved in 70% ethanol.

After arrival, the lung tissue was refixed in 4% formalin solution and used as starting material. Eight blocks of each lung were selected by smooth fractionator method as described earlier and were cut in cubes of 10 × 10 × 10 mm. One cube of each block was selected for embedding. The eight cubes of one lung were randomly placed in the embedding molds and embedded in methylmetacrylate. Briefly, tissue was washed first in 100% ethanol using a gradient series, followed by Histoclear (Merck, Schaffhausen, Switzerland) and by a solution of four parts of methylmetacrylate and one part of dibutyl phthalate (both Fisher Scientific AG, Reinach, Switzerland). The resin was polymerized using 2–3% dicumyl peroxide (Merck, Schaffhausen, Switzerland). Four cubes per mold were embedded. Due to its geometry, it does not result a completely random orientation of the tissue blocks, but the structure of interest, capillary layers of the alveolar septa, can be assumed to being isotropically (18–21). Sections (2 µm) were cut and stained with Goldner staining, following an adapted protocol due to the thinness of the sections. The incubations with Ponceau acid fuchsin and phosphomolybdic acid-orange G and light green were extended by 30 min each (22).

### Stereology

**Figure 1.**
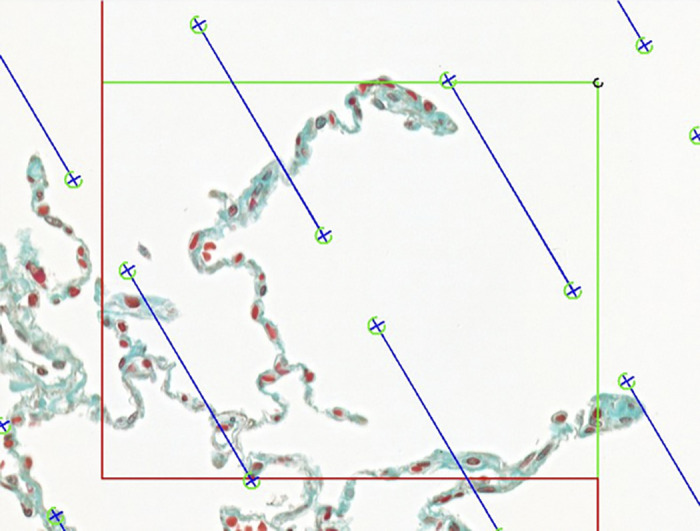
Counting frame used for the determination of the microvascular maturation quotient.

Stereological counting was performed with the software newCast from Visopharm (Hoersholm, Denmark). After the import of the digitalized slides (two per lung), the regions of interest were chosen systematically and uniformly randomly by the software. A counting frame was superimposed onto the selected regions of interest (Fig. 1). The two counting events were defined as linear intercepts of these lines with mature or immature alveolar septa, respectively. As previously described, mature alveolar septa were defined as septa with a single-layered capillary network and immature alveolar septa were defined as septa with double-layered capillary networks. For each of the described counting categories, at least 200 events per lung were registered except for immature alveolar septa in the two juvenile samples, due to their very rare appearance (115 and 117 counting events, respectively).

To evaluate the data, the microvascular maturation quotient (MVM-quotient) was defined as

$$\frac{\mathrm{Number}\;\mathrm{of}\;\mathrm{mature}\;\mathrm{intersections}}{\mathrm{Number}\;\mathrm{of}\;\mathrm{mature}\;\mathrm{intersections}+\mathrm{Number}\;\mathrm{of}\;\mathrm{immature}\;\mathrm{intersections}}$$

The number of alveoli as well as age, sex, and growth data for each subject were adopted from the results of Herring et al. (7).

### Statistics

Age and number of alveoli were used as predictors against the measured variables. Three functions were considered for each outcome: linear, exponential, or logarithmic. The adjusted (Adj) R^2^ value (coefficient of determination) was used to identify the best fitting model. If the model is linear, it will increase or decrease in a uniform manner. If it is exponential, the model will initially increase or decrease slowly, but will get a faster increase/decrease in the course.

## RESULTS

### Study Population

Table 1 lists the sex, age, and growth data, as well as the number of alveoli, measured by Herring et al. (7) of each subject studied. There were 12 subjects (3 females, 9 males) with a range in age from 24 days to 15 yr and 11 mo. There were no pulmonary lesions or disorders noted at the autopsy. At least two subjects were born prematurely. Nevertheless, no correction for gestational age was applied to interpret the results. In this point, we followed Herring et al. (7) to keep the comparability to the previously obtained dataset. To our best knowledge, none of the subjects encountered any kind of lung diseases like bronchopulmonary dysplasia, acute respiratory distress syndrome, or persistent pulmonary hypertension.

**Table 1.** Age, sex, growth data, and gestational age of each subject

Age	Sex	Height, cm	Weight, kg	Weeks of pregnancy	Number of Alveoli
24 days	M	54.5	4.85	Unknown	
1 mo 3 days	M	56	4.64	39.1	106,514,687.5
1 mo 25 days	M	57	4.66	40	143,240,259.7
2 mo 1 days	M	58.5	5.76	Unknown	
2 mo 9 days	M	52	3.92	33.4	136,367,142.9
3 mo 6 days	M	66	7.3	41.6	204,779,571.4
4 mo 5 days	M	64.5	6.6	40	273,611,688.3
4 mo 16 days	F	62	6.24	36	188,067,551
5 mo	F	68.5	7.7	40.4	161,093,571.4
2 yr 6 mo	M	96.5	14.1	Unknown	430,630,633.9
15 yr	F	165	65.3	NR	583,899,357.1
15 yr 11 mo	M	150	53.5	NR	486,792,250

F, female; M, male; N_alveoli_, Number of alveoli in the lung, results from Herring et al. (7); NR, not relevant.

#### MVM-quotient.

**Figure 2.**
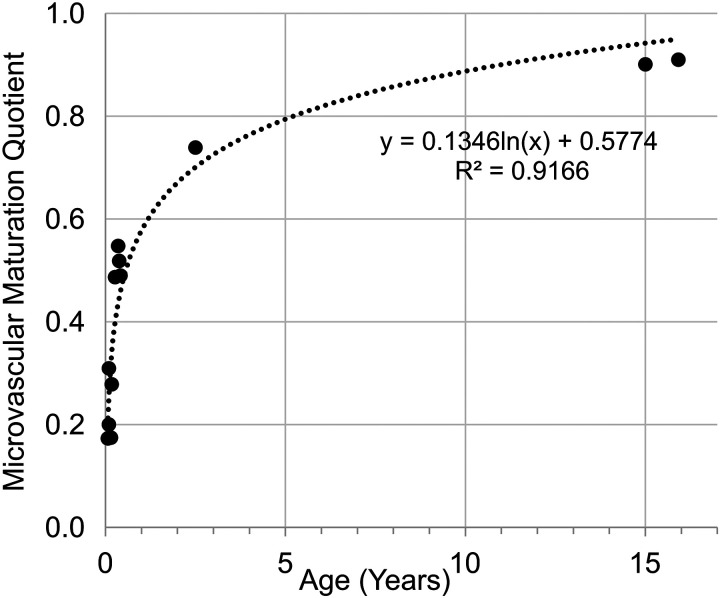
Microvascular maturation of the alveolar capillaries. The MVM-quotient versus age (years). Dotted line, the trendline of MVM-quotient versus age is given by a logarithmic function. *n* = 12, 9 males and 3 females. MVM, microvascular maturation.

Table 2 shows the raw values of the MVM-quotient for age. MVM-quotient versus age showed a logarithmic dependency with an Adj R^2^ value of 0.92 of the trendline, with a fast increase in the first phase to the age of 2 yr. Subsequently, the increase tapers off but continues to grow, never reaching the value of one (Fig. 2). The trendlines formula of MVM-quotient versus age is f(x)= 0.1373ln(x) + 0.2315.

**Table 2.** Age, sex, and microvascular maturation quotient of each subject

Age	Sex	MVM-Quotient
24 days	M	0.172938351
1 mo 3 days	M	0.2
1 mo 25 days	M	0.174001354
2 mo 1 days	M	0.23
2 mo 9 days	M	0.30926431
3 mo 6 days	M	0.48639456
4 mo 5 days	M	0.547339946
4 mo 16 days	F	0.51816239
5 mo	F	0.49
2 yr 6 mo	M	0.74
15 yr	F	0.90086207
15 yr 11 mo	M	0.91

F, female; M, male; MVM, microvascular maturation.

**Figure 3.**
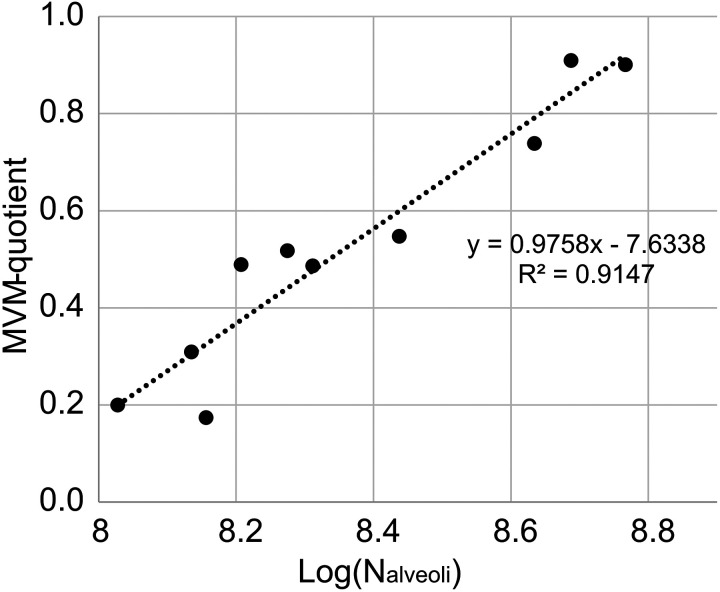
Comparison between microvascular maturation and alveolarization. The data describing the number of alveoli (N_alveoli_) are taken from Herring et al. (7). Dotted line, MVM-quotient versus Log (N_alveoli_) is plotted in a linear function (Table 2). *n* = 12, 9 males and 3 females. MVM, microvascular maturation.

The MVM-quotient was also compared with the logarithmic number of alveoli (Fig. 3) measured in the study of Herring et al. (7). The figure shows a linear dependency of the inserted trendline between these parameters with an Adj R^2^ value of 0.91. The trendline’s function is f(x)= 0.9758x − 7.6338.

## DISCUSSION

In mice and rats, it was shown by stereological estimations that the maturation of the alveolar septa, in particular the maturation of the microvasculature, progresses hand in hand with alveolarization (13, 15, 16). In humans, only morphological observations exist, e.g., studies by Burri et al. (14) and Caduff et al. (1). To our best knowledge, human microvascular maturation has never been studied by stereological estimations due to the lack of the appropriate samples and methods. Therefore, based on tissue from Herring et al. (7), a stereological estimation on 12 human lungs aged 24 days to 16 yr was performed to study the relation between age and microvascular maturation. This correlation is not linear, rather it is a logarithmic dependency, as the trendline in Fig. 2 shows. This means that the bulk of microvascular maturation takes place early in human life. Roughly after 7 mo, half of the microvascular maturation is completed (Fig. 2). Furthermore, it is possible to calculate the potential endpoint of microvascular maturation in human lungs. Based on the results, the calculated potential endpoint of the microvascular maturation is at the age of ∼22.5 yr.

Based on morphological observations, the endpoint of microvascular maturation was described at the age between the second and third year of life (12, 14). The stereological estimation now shows that it continues until young adulthood. As supposed in a recent review of lung development by one of the authors (J.C.S.) (13), alveolarization and microvascular maturation are processes that take place in parallel. The formation of the secondary alveolar septum explains this thesis, because every secondary septum possesses, at least for a short time, a double-layered capillary network that fuses during microvascular maturation to a single-layered capillary network. This leads to the fact that as long as alveolarization takes place, microvascular maturation will also take place (13).

Herring et al. (7) showed in a foregoing study with the same lung tissue as used in this study that alveolarization lasts until young adolescence. It was shown that the number of alveoli continues to rise until young adulthood, but at a reduced rate after 2 yr (7). Therefore, this study also compared the logarithmic number of alveoli of the study of Herring et al. (7) and the microvascular maturation quotient (Fig. 3). The linear correlation between these parameters verifies the hypothesis that alveolarization and microvascular maturation in human lungs are processes that take place in parallel (13). This means that as long as new alveoli are built and the number of alveoli increases, MVM will also happen in the human lung, supporting the assumption of increased plasticity of the human lung and the potential to recover from early childhood lung diseases.

Due to the limited availability of suitable human tissue, we were not able to choose the time points resulting in a quite small number of time points older than half a year. Therefore, it is not possible to confirm that microvascular maturation in human lungs continues until the age of around 22.5 yr as calculated. Inevitably, the question is whether microvascular maturation in the human lung comes to an end or not, because our data never show a microvascular maturation quotient of 1 which is fully mature. The same is true for mice and rats (15, 16). This result may be explained by a possible process of regeneration and conversion of alveolar septa, meaning that there may be a constant loss and rebuilding of alveoli over the entire adult life. If so, microvascular maturation continues for a lifetime and the maturation quotient will never reach the value of 1 because every newly formed septum is immature. This hypothesis is supported by a case study of Butler et al. (23), where a postinterventional growth of the human lung is described in an adult patient after lobectomy. Clinically, it means that the lung has the potential to regenerate even in adult age. However, it appeared to be depending on the kind of insult and at which time point of life it takes place. After lobectomy at relatively young age, regeneration appears to be possible (23). Early premature-born patients who develop “bronchopulmonary dysplasia,” also called “prematurity-associated obstructive lung disease,” show lifelong ventilation abnormalities and abnormal lung microstructure. Early prematurity alone, without the development of this disease, causes non or only little long-term lung abnormalities. There is evidence that bronchopulmonary dysplasia causes persistent neutrophilic airway inflammation and oxidative stress (24). Therefore, the observed lifelong reduction of lung function may be due to the persistence of the disease and not due to missing of pulmonary regeneration. In the case of premature-born babies without bronchopulmonary dysplasia, it may be speculated that these patients either did not encounter significant persistent lung damage and/or were able to activate pulmonary regeneration (25, 26).

## DATA AVAILABILITY

Data will be made available upon reasonable request.

## GRANTS

This work was supported by the National Institutes of Health Grants P01-ES-0628 and OD-011107, as well as by the Swiss National Science Foundation Grants 31003 A-109874 and 310030_175953.

## DISCLOSURES

No conflicts of interest, financial or otherwise, are declared by the authors.

## AUTHOR CONTRIBUTIONS

J.C.S. conceived and designed research; L.S. and D.M.H. performed experiments; L.S. and J.C.S. analyzed data; L.S. and J.C.S. interpreted results of experiments; L.S. and J.C.S. prepared figures; L.S. drafted manuscript; L.S., D.M.H., and J.C.S. edited and revised manuscript; L.S., D.M.H., and J.C.S. approved final version of manuscript.
